# The clinical application value of mixed‐reality‐assisted surgical navigation for laparoscopic nephrectomy

**DOI:** 10.1002/cam4.3189

**Published:** 2020-06-15

**Authors:** Guan Li, Jie Dong, Jinbao Wang, Dongbing Cao, Xin Zhang, Zhiqiang Cao, Guangming Lu

**Affiliations:** ^1^ Department of Radiology Jinling Hospital Nanjing Medical University Nanjing China; ^2^ Department of Urology General Hospital of Northern Theater Command Shenyang China; ^3^ Department of Urology Jinling Hospital Nanjing Medical University Nanjing China; ^4^ Department of Radiology General Hospital of Northern Theater Command Shenyang China; ^5^ Department of Urology Cancer Hospital of China Medical University Shenyang China; ^6^ Department of Radiology The First Affiliated Hospital of China Medical University Shenyang China

**Keywords:** computer tomography, laparoscopic nephrectomy, mixed reality, renal cell carcinoma, surgical navigation

## Abstract

**Purpose:**

Laparoscopic nephrectomy (LN) has become the preferred method for renal cell carcinoma (RCC). Adequate preoperative assessment or intraoperative navigation is key to the successful implementation of LN. The aim of this study was to evaluate the clinical application value of mixed‐reality–assisted surgical navigation (MRASN) in LN.

**Patients and Methods:**

A total of 100 patients with stage T1N0M0 renal tumors who underwent laparoscopic partial nephrectomy (LPN) or laparoscopic radical nephrectomy (LRN) were prospectively enrolled and divided into a mixed‐reality‐assisted laparoscopic nephrectomy (MRALN) group (n = 50) and a non–mixed‐reality‐assisted laparoscopic nephrectomy (non‐MRALN) group (n = 50). All patients underwent renal contrast‐enhanced CT scans. The CT DICOM data of all patients in the MRALN group were imported into the mixed‐reality (MR) postprocessing workstation and underwent holographic three‐dimensional visualization (V3D) modeling and MR displayed, respectively. We adopted the Likert scale to evaluate the clinical application value of MRASN. The consistency of evaluators was assessed using the Cohen kappa coefficient (k).

**Results:**

No significant differences in patient demographic indicators between the MRALN group and the non‐MRALN group (*P *> .05). The subjective score of MRASN clinical application value in operative plan formulation, intraoperative navigation, remote consultation, teaching guidance, and doctor‐patient communication were higher in the MRASN group than in the non‐MRASN group (all *P* < .001). There were significantly more patients for whom LPN was successfully implemented in the MRALN group than in the non‐MRALN group (82% vs 46%, *P* < .001). The MRALN group had a shorter operative time (OT) and warm ischemia time (WIT) and less estimated blood loss (EBL) than the non‐MRALN group (all *P* < .001).

**Conclusion:**

MRASN is helpful for operative plan formulation, intraoperative navigation, remote consultation, teaching guidance, and doctor‐patient communication. MRALN may effectively improve the successful implementation rate of LPN and reduce the OT, WIT, and EBL.

## Graphical abstract

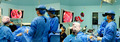

## 1 INTRODUCTION

In recent years, with the development of minimally invasive surgery, laparoscopic nephrectomy (LN) has become the preferred method for stage T1N0M0 renal cell carcinoma (RCC).^1^ When RCC occurs in special locations, such as totally intrarenal tumors (TIT) or hilar tumors, we discovered that there are cases in which the tumor may not be found during the operation, which causes difficulty in performing LN.^2^ At present, we often used CT images as a routine method for preoperative observation or evaluation of renal tumors.^3^ Traditional CT images are usually two‐dimensional (2D) images, which challenges the surgeon's three‐dimensional (3D) space sense.

With the development of holographic visualization technology and the emergence of the Fifth‐Generation (5G) network, which have laid a foundation for the advancement of novel surgical navigation,^4^, ^5^ currently, mixed‐reality‐assisted surgical navigation (MRASN) can provide surgeons with a new perspective or strategy. Mixed‐reality (MR) technology introduces virtual scenes to the real environment and establishes an interactive feedback information loop between the virtual world, the real world, and the user.^6^ Verhey et al^7^ introduced the concept that MR technology can be helpful for preoperative operation plan formulation by a 360 degree panoramic display of a renal tumor without a dead angle, which contributes to improving preoperative planning efficiency and has better familiarity with patient anatomy. Yoshida et al^8^ reported that real‐time imaging of intraoperative MRASN can be conducive to the tracking and location of tumors and improve the accuracy of renal tumor resection. Condino et al^9^ explored whether MR technology can be helpful for teaching practice. Through an MR preoperative simulation training system, the operation efficiency and accuracy of surgeons are improved. Furthermore, MR technology also helps patients understand the operation process and facilitates doctor‐patient communication.^10^, ^11^

When MRASN is applied to LN, namely, in mixed‐reality–assisted laparoscopic nephrectomy (MRALN), surgeons can not only clearly display the renal tumor (including shape, size, location, number, etc) and the relationship of the tissue structure around the renal tumor (such as the renal blood vessels, collection system, adjacent organs, and adherent perinephric fat [APF] area), but also easily and accurately locate the renal tumor both before and during the operation. APF, which is also called “perinephric sticky fat (PSF)”, affects the detachment of renal tumors and may increase the risk of operative bleeding, as well as the operative time (OT) and surgical conversion rate.^12^, ^13^ The aim of this study was to evaluate the clinical application value of MRASN and the application of MRALN to RCC.

## 2 PATIENTS AND METHODS

### 2.1 Study design and population

Patients (n = 100; 67 males and 33 females, with a median age of 56.7 ± 13.8 years and age range of 33‐79 years) with stage T1N0M0 renal tumors who underwent laparoscopic partial nephrectomy (LPN) or laparoscopic radical nephrectomy (LRN) in our institution were reviewed prospectively between January 2019 and October 2019. One hundred patients were divided into two groups based on whether they underwent MR, namely, the MRALN group (n = 50 cases) and the non‐MRALN group (n = 50 cases). Two senior surgeons (each surgeon had completed at least 500 cases) on the same team performed all 100 operations. One middle radiologist (with ten years of experience in 3D reconstruction) performed all holographic three‐dimensional visualization (V3D) modeling, and the mean time to complete each 3D model was approximately 30 minutes. Our institutional review board endorsed the study as a prospective study, and all patients gave informed consent to participate in the investigation.

### 2.2 Inclusion and exclusion criteria

The inclusion criteria were as follows: (I) all patients underwent renal contrast‐enhanced CT scanning; (II) all patients had a stage T1 renal tumor; (III) all renal tumors had a RENAL nephrometry score^14^ ≥7; (IV) all operations were performed by two senior surgeons; and (V) surgical records of the presence and area of APF were available. The exclusion criteria were as follows: (I) CT contrast agent allergy; (II) severe cardiovascular and cerebrovascular diseases; (III) severe kidney dysfunction; and (IV) severe renal artery stenosis or occlusion.

### 2.3 CT DICOM data acquisition

**FIGURE 1:**
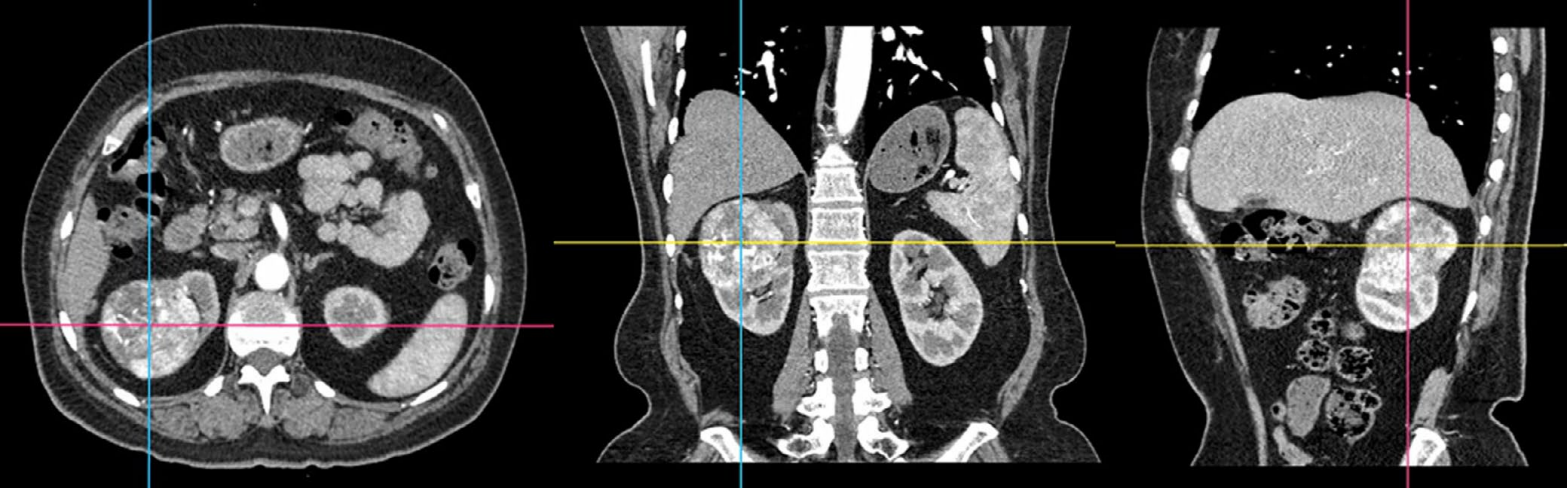
CT DICOM data acquisition and multiplanar reconstruction (MPR)

A renal contrast‐enhanced scan was performed by a CT scanner (SOMATOM Definition Flash, Siemens Healthcare, Forchheim, Germany). The scanning parameters were as follows: tube voltage and tube current (tube A 100 kVp, 149 eff.mAs and tube B Sn 140 kVp, 117 eff.mAs); detector configuration, 64 × 0.6 mm; gantry rotation time, 0.5 seconds; and pitch, 1.2. All scans adopted automatic current modulation (CareDose4D; Siemens Healthcare). The contrast agent iohexol (Omnipaque 350, GE Healthcare) was injected into the anterior cubital vein with a dual‐cylinder high‐pressure syringe (Urich Medical) at a rate of 4 mL/s and a dose of 1.5 mL/kg, with a total volume of approximately 60‐80 mL. The region of interest (ROI) was placed at the branch level of the abdominal aorta and renal artery by using bolus tracking software to trigger. When the threshold reached 100 HU, a renal arterial phase CT scan was performed after a delay of 7 seconds, followed by a delay of 40 seconds and 5 minutes for renal venous‐phase and delayed‐phase scanning, respectively. All images adopted a thickness of 0.75 mm and an interval of 0.5 mm with a kernel of Q30f to finish the reconstruction (Figure 1).

### 2.4 Holographic V3D modeling and MR displayed

**FIGURE 2:**
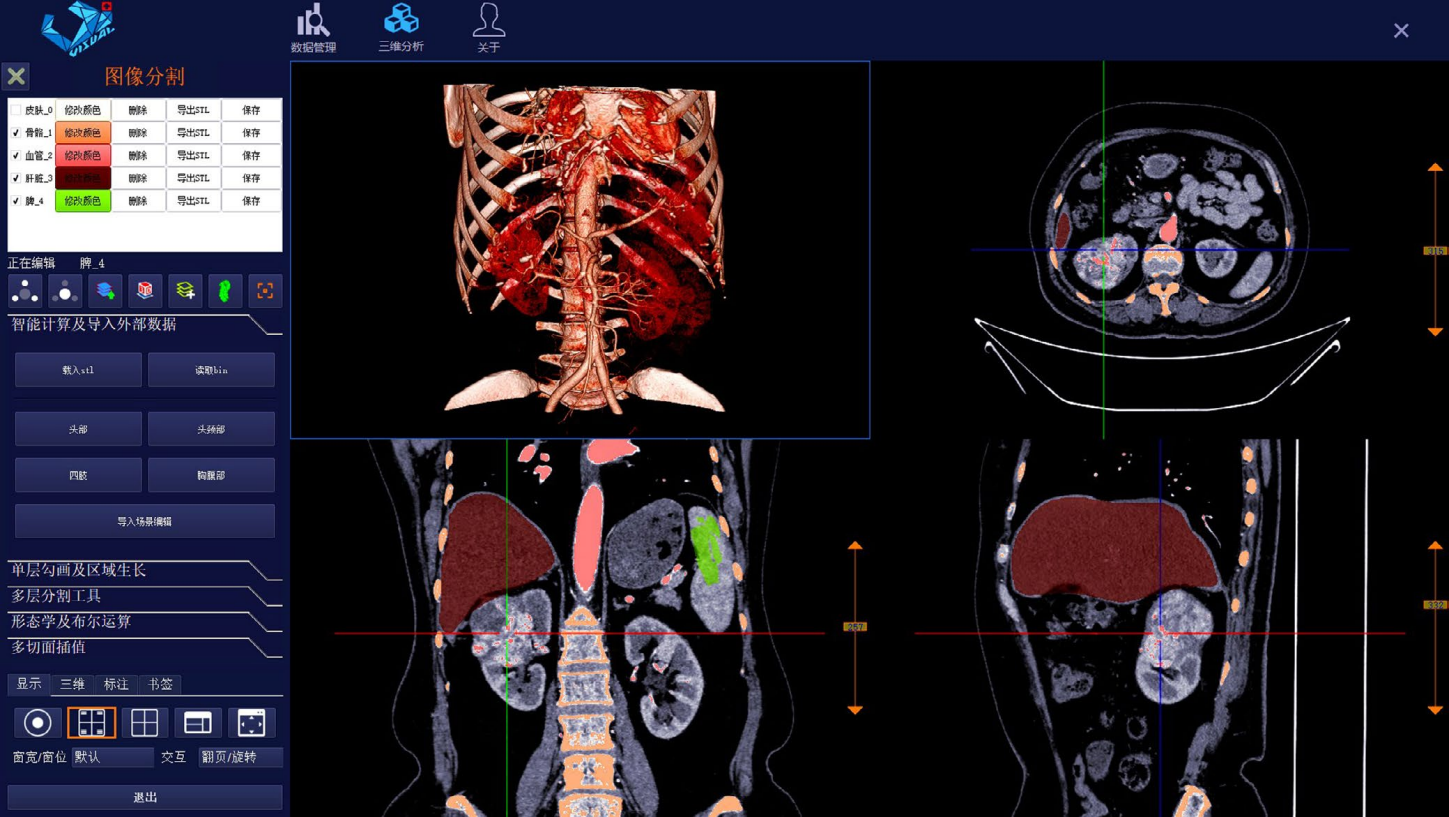
Holographic V3D modeling software interface

**FIGURE 3:**
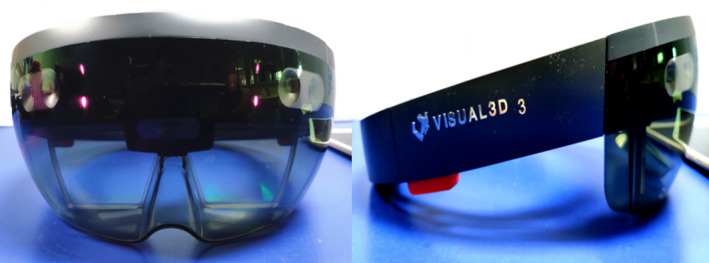
HoloLens MR‐HMD

CT DICOM data of the MRALN group were input into holographic V3D modeling software (Visual3d Medical Technology Development Co., Ltd. (VISUAL), Beijing, China). Visual edge detection, automatic segmentation extraction, and registration steps were sequentially applied to target organs, tissues, and tumors. For areas that were not automatically recognized or had segmented blurred, we selected manual ROI draw compensation. We removed all irrelevant or unconnected parts (such as the CT bed plate, foreign body in vitro, retained tube shadow, and etc) and merely retained the kidney, tumor, renal vessels, renal collection system, skin, skeleton, liver, and spleen. For the APF region, we chose manual ROI drawing to achieve segmentation. All target organs, tissues, and tumors were expanded, corroded, and smoothed in turn, and saved as the standard template library (STL) files separately. Then, all STL files were imported into scene editing mode. In this model, we could adjust the color and transparency of target organs, tissues, and tumors to obtain the best holographic V3D modeling effect (Figure 2). V3D modeling data were imported into the laparoscopic video system or MR equipment (using the HoloLens head‐mounted display (HMD)) (Figure 3).

### 2.5 Operative method and MRASN

**FIGURE 4:**
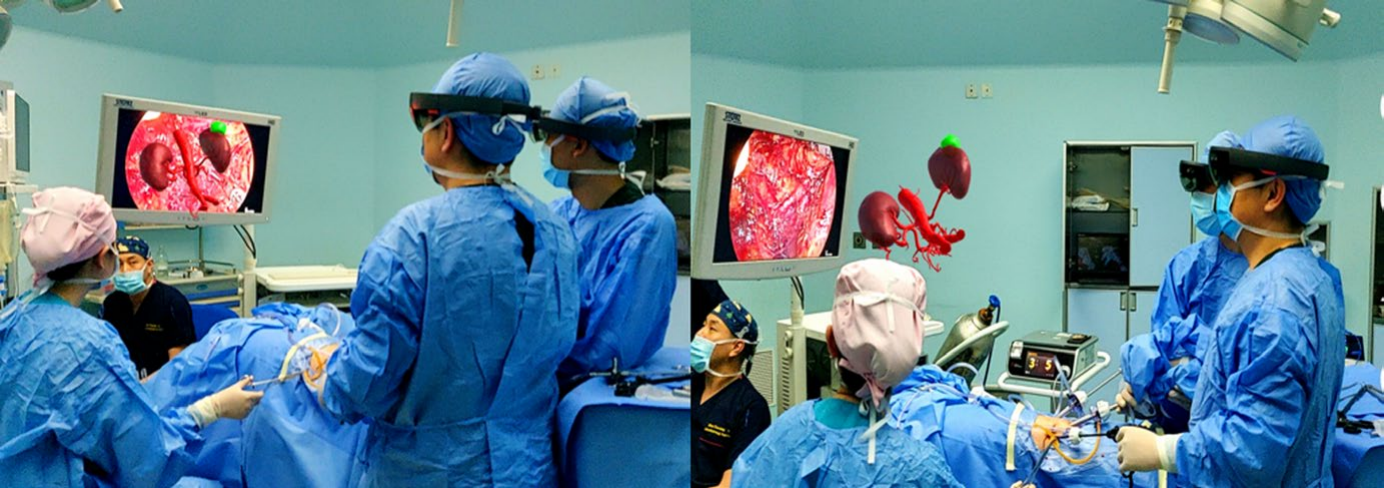
Application scenarios of MRASN in surgery

Laparoscopic partial nephrectomy was performed under standard general anesthesia. The patient's body position was obtained by a retroperitoneal approach. First, the retroperitoneal space was established using the open Hasson technique.^15^ A homemade balloon was placed in the retroperitoneal cavity, and then inflated with 600 mL of CO_2_. Second, three channels were established: hole A: a 10.0‐mm diameter trocar was placed approximately 2.0 cm above the iliac crest of the axillary midline; hole B: a 12.0‐mm diameter trocar was placed approximately 1.0 cm under the 12th costal margin of the axillary front; hole C: a 5.0‐mm‐diameter trocar was placed approximately 2.0 cm under the 12th costal margin of the axillary posterior line. Third, the perirenal fascia and renal fat sac were dissected longitudinally and dissected by an ultrasonic scalpel to fully expose the renal tumor and the renal parenchyma around the tumor. Fourth, intraoperative navigation was performed. We imported MR results and laparoscopic video stream into a new monitor (with a video capture card, such as the TiePro display function of the robotic surgery window), and fusion was performed in the new monitor or introduced into HoloLens MR‐HMD (Figure 4). The “vascular bifurcation labeling” technology (namely, registering the bifurcation of the abdominal aorta and the renal artery) was adopted to achieve the registration, fusion, tracking, and real‐time comparison between the MR image and intraoperative real scene. The surgeon can use the functions of rotation, splitting, hiding, and scaling of the MR image to ensure accurate resection and the safety of the operation. Fifth, renal tumor resection was performed. The blood supply artery of the tumor was blocked by bulldog clamp, and the renal parenchyma approximately 0.5 cm from the edge of the tumor was removed along the tumor capsule. Sixth, the V‐lock line combined with the Hem‐o‐lok clip was adopted to suture the defects of the renal parenchyma. Finally, the bulldog clamp was removed, and the renal blood supply was restored.

### 2.6 Subjective evaluation of MRASN

**FIGURE 5:**
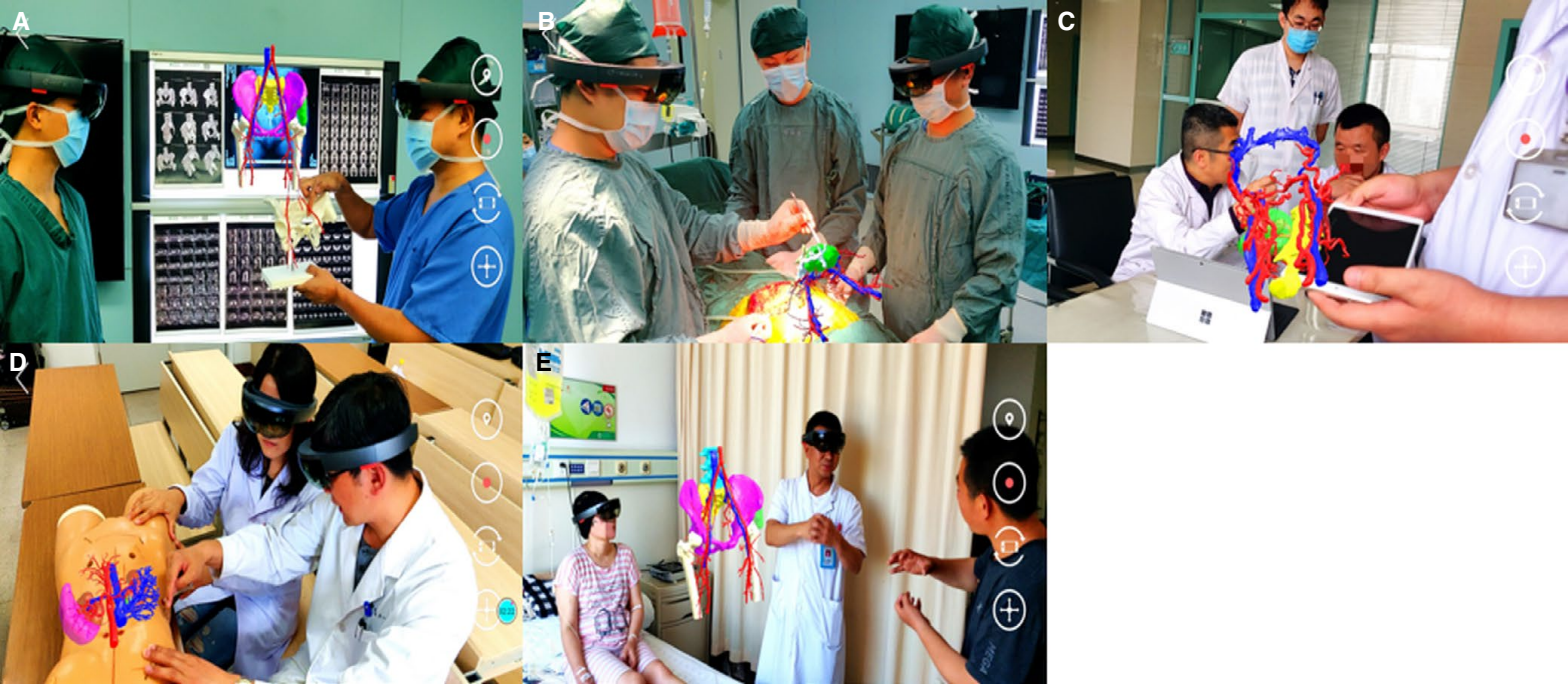
MRASN clinical application value display. A, Operative plan formulation. B, Intraoperative navigation display. C, Remote consultation. D, teaching guidance. E, Doctor‐patient communication

Six surgeons (two senior, two middle, and two junior) were selected to evaluate the clinical application value of MR by the HoloLens HMD mode. Two surgeons with the same professional title scored the MR results of the same patient. When the scoring results showed significant inconsistencies, the final scoring results were given after negotiation. The surgeon's experience was categorized according to the number of LNs performed: junior (during the first 100 cases), middle (between 100 and 300 cases), and senior (after the surgeon had completed 300 cases and above). Descriptive analysis of the results was performed for the Likert‐type questionnaire's items with a 5‐point scale, as follows: 10 points: strongly agree, 7‐9 points: agree, 4‐6 points: neither agree nor disagree, 1‐3 points: disagree, and 0 points: strongly disagree. The questionnaire's items include the role of MR in i) operative plan formulation, ii) intraoperative navigation, iii) remote consultation, iv) teaching guidance and v) doctor‐patient communication (Figure 5). In the non‐MRASN group, we chose the same patient's CT results to score the above five items. Six surgeons evaluated the MR results (MRASN group) and CT results (non‐MRASN group) of all patients subjectively in the above five aspects to calculate the mean ± standard deviation (SD).

### 2.7 Statistical analysis

Statistical analyses were performed with IBM SPSS Statistics (version 22.0.0, Armonk). The measurement data of the two groups are expressed as the mean ± standard deviation (SD). Mean comparisons between two independent samples were performed with the two‐sample *t* test. The counting data of the two groups were examined by the chi‐square test. The hierarchical data of the two groups were analyzed by the Wilcoxon test. The Pearson chi‐squared test was used to test for correlations between the MRALN and non‐MRALN groups. A *P* value <.05 was considered statistically significant. For the consistency of MR clinical application value evaluation between the two surgeons of the same rank, we applied the kappa test, which could be interpreted as follows: 0.80 ≤ k < 1.00, very good agreement; 0.60 ≤ k < 0.80, good agreement; 0.40 ≤ k < 0.60, moderate agreement; k < 0.40, poor agreement.

## 3 RESULTS

### 3.1 Patient characteristics and clinical information

Table 1 summarizes the demographic characteristics of the cohort. The results showed that there were no significant differences in mean age, height, weight, sex, body mass index (BMI), preoperative serum creatinine (Scr), preoperative estimated glomerular filtration rate (eGFR), RENAL score, and Mayo Adhesive Probability (MAP) score^16^ between the MRALN group and the non‐MRALN group (all *P *> .05).

**TABLE 1:** Comparison of demographic characteristics between the MRALN group and non‐MRALN group

Variable	MRALN group (n = 50)	Non‐MRALN group (n = 50)	*P* value
Mean age (y)	54.3 ± 12.1	56.9 ± 14.7	.337
Height (cm)	170.3 ± 10.8	173.4 ± 11.4	.166
Weight (kg)	82.5 ± 11.4	85.8 ± 12.7	.175
Sex (%)			.398
Male	31 (62%)	35 (70%)	
Female	19 (38%)	15 (30%)	
BMI (kg/m^2^)	28.4 ± 1.4	28.5 ± 1.6	.740
Preoperative Scr (μmol/L)	76.4 ± 7.5	78.1 ± 8.4	.288
Preoperative eGFR (mL/min)	107.9 ± 11.1	109.2 ± 15.5	.631
RENAL score	8.9 ± 1.9	8.4 ± 1.6	.158
MAP score	1.9 ± 0.7	1.8 ± 0.4	.383

### 3.2 Operative outcome

One hundred patients successfully completed the operation. No cases were converted into open surgery. According to the application of MR technology, RENAL nephrometry score, and MAP score results, there were 41 cases of LPN, 9 cases of LRN, 36 cases of T1a, and 14 cases of T1b in the MRALN group, and 23 cases of LPN, 27 cases of LRN, 38 cases of T1a, and 12 cases of T1b in the non‐MRASN group (Table 2). Postoperative pathology results showed that the MR group had 39 cases of clear‐cell renal cell carcinoma (ccRCC), 5 cases of papillary renal cell carcinoma (pRCC), 4 cases of chromophobe renal cell carcinoma (chRCC), and 2 cases of multilocular cystic renal neoplasm of low malignant potential (MCRNLMP). The non‐MR group had 37 cases of ccRCC, 4 cases of pRCC, 3 cases of chRCC, 2 cases of Xp11 translocation renal cell carcinoma (TRCC), 2 cases of renal medullary carcinomas (RMCs) and collecting duct carcinomas (CDCs), 1 case of MCRNLMP, and 1 case of renal oncocytoma (RO).

**TABLE 2:** Comparison of operative and perioperative outcomes between the MRALN and non‐MRALN groups

Parameter	MRALN group (n = 50)	Non‐MRALN group (n = 50)	*P*value
OT, min	60.7 ± 10.4	98.4 ± 11.7	<.001
WIT, min	12.5 ± 1.2	20.3 ± 0.9	<.001
EBL, mL	15.5 ± 9.4	45.9 ± 10.1	<.001
Hospital stay, days	6.8 ± 1.0	7.0 ± 0.9	.296
Preoperative creatinine, mol/L	92.4 ± 11.5	88.5 ± 13.4	.121
Postoperative creatinine, mol/L	106.3 ± 12.4	111.5 ± 14.7	.059
LPN conversion to LRN, No. (%)	2 (4)	4 (8)	.402
Postoperative complication, No. (%)	1 (2)	3 (6)	.312
Underwent LPN, No. (%)	41 (82)	23 (46)	<.001
T1a stage, No. (%)	36 (72)	38 (76)	.651
T1b stage, No. (%)	14 (28)	12 (24)	.651

Abbreviations: EBL, estimated blood loos; OT, operative time; WIT, warm ischemia time.

### 3.3 Objective evaluation

Table 2 summarizes the operative and perioperative outcomes in the cohort. There were significantly more patients who underwent LPN in the MRASN group than in the non‐MRASN group (82% vs 46%, *P* < .001). Patients in the MRALN group had a significantly shorter OT (60.7 ± 10.4 vs 98.4 ± 11.7 minutes, *P* < .001) and warm ischemia time (WIT) (12.5 ± 1.2 vs 20.3 ± 0.9 minutes, *P* < .001) and less estimated blood loss (EBL) (15.5 ± 9.4 vs 45.9 ± 10.1 mL, *P* < .001) than did patients in the non‐MRALN group. No significant difference was found in the stage of renal tumor, LPN conversion to LRN, hospital stay, postoperative complications, and preoperative or postoperative creatinine (all *P *> .05).

### 3.4 Holographic V3D image and MR displayed

**FIGURE 6:**
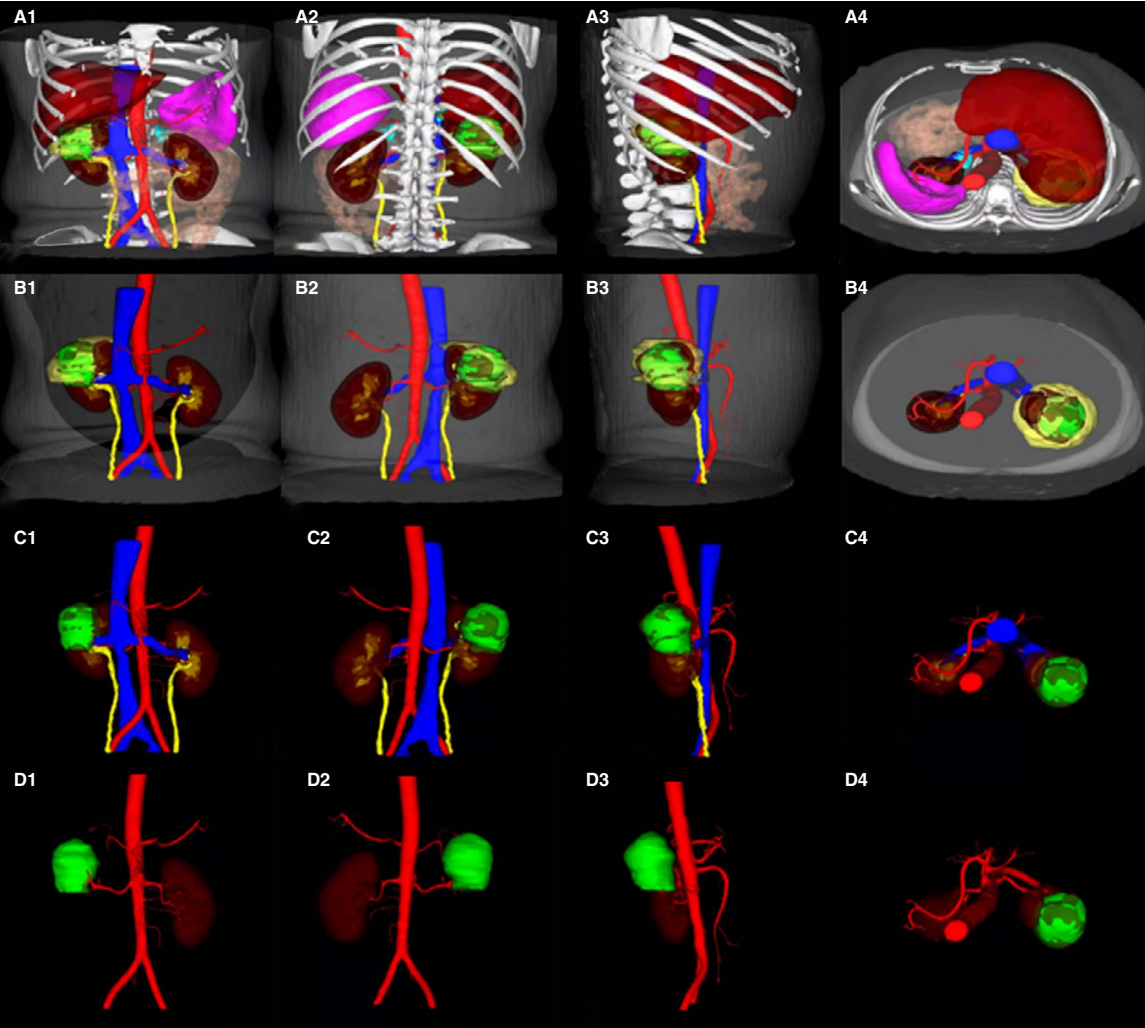
Holographic V3D image displayed (from outside to inside, gray: skin, white: bone, pink: intestine, red: artery, blue: vein, brownish red: liver, purple pink: spleen, light yellow: APF, vermilion: kidney, yellow: collecting system, turquoise blue: adrenal gland and green: tumor). A1‐4: multidirectional and multiangle display of abdominal organs and tissues; B1‐4: multidirectional and multiangle display of the kidney, renal tumor, renal arteriovenous and collecting system and APF; C1‐4: multidirectional and multiangle display of kidney, renal tumor, renal arteriovenous and collecting system; D1‐4: multidirectional and multiangle display of the renal tumor and arterial blood supply

**FIGURE 7:**
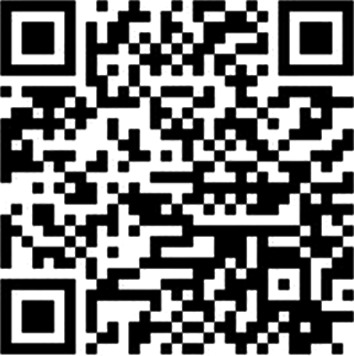
Scan the two‐dimensional code to view the holographic V3D image on your smartphone

**FIGURE 8:**
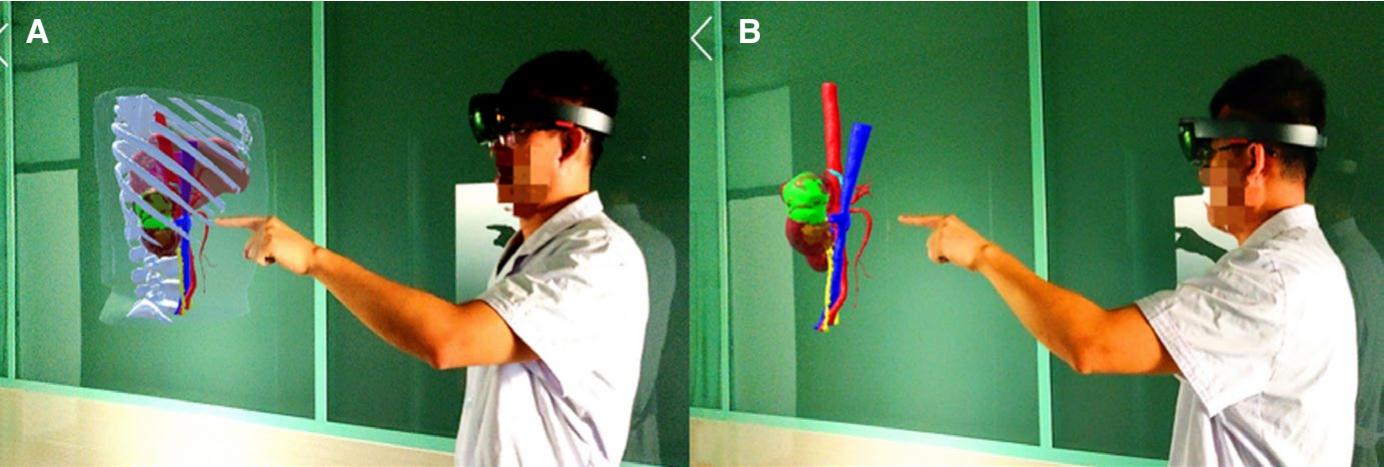
MR displayed under HoloLens HMD. MR display result shooting from a third‐party perspective

In the MR group, 50 patients underwent holographic V3D image acquisition and had a completed MR display. The renal tumor and peritumoral tissue structure (including kidney, renal arteriovenous, collecting system, adrenal gland, liver, spleen, intestine, and bones) was clearly and selectively observed. The area of APF was displayed accurately (Figures 6, 7, 8).

### 3.5 Subjective evaluation

Table 3 shows the subjective scores of the MRASN clinical application value between the MRASN and non‐MRASN (conventional CT image) groups. Fifty patients in the MR group were selected to evaluate the clinical application value of MRASN and conventional CT images. The scores of operative plan formulation, intraoperative navigation, remote consultation, teaching guidance, and doctor‐patient communication were higher in the MRASN group than in the non‐MRASN group (8.6 ± 0.6 vs 4.1 ± 1.1, 8.6 ± 0.6 vs 2.3 ± 0.9, 8.4 ± 0.6 vs 3.7 ± 1.4, 8.6 ± 0.7 vs 3.9 ± 1.5, 8.5 ± 0.7 vs 3.9 ± 1.3, all *P* < .001). Interobserver agreement was reached in good consistency (two senior surgeons, k = 0.82; two middle surgeons, k = 0.84; two junior surgeons, k = 0.85).

**TABLE 3:** Comparison of subjective scores of the clinical application value between the MRASN and non‐MRASN groups

Score	MRASN group (n = 50)	Non‐MRASN group (n = 50)	*P*value
Operative plan formulation	8.6 ± 0.6	4.1 ± 1.1	<.001
Intraoperative navigation	8.6 ± 0.6	2.3 ± 0.9	<.001
Remote consultation	8.4 ± 0.6	3.7 ± 1.4	<.001
Teaching guidance	8.6 ± 0.7	3.9 ± 1.5	<.001
Doctor‐patient communication	8.5 ± 0.7	3.9 ± 1.3	<.001

## 4 DISCUSSION

MR is a novel digital holographic imaging technology that combines the advantages of virtual reality (VR) and augmented reality (AR).^17^ MR integrates a virtual model drawn by a computer into the real‐world scene viewed by users, which allows the surgeon to observe the lesion from different angles and different sites. MR can be registered and fused with the patient's body, and the surgeon can even enter the image to observe the lesions. At present, MRASN mainly has two modes: one is applied to open nephrectomy, namely, nonlaparoscopic nephrectomy, in which the surgeons use HoloLens MR‐HMD to precisely fuse virtual hologram images with the surgical area to achieve the purpose of precise positioning and precise surgery^10^; the other is to precisely fuse the preoperative holographic V3D image with the field image under the laparoscope monitor,^18^ to achieve real‐time comparison and tracking and guide the operation safely and smoothly. We demonstrated the clinical application value of MRASN in operative plan formulation, intraoperative navigation, remote consultation, teaching guidance, and doctor‐patient communication.

For a stage T1 renal tumor (RENAL nephrometry score ≥7) or renal tumor occurring in the isolated kidney, LPN could protect renal function better than LRN.^14^ Due to the application of MR technology, surgeons could more comprehensively analyze renal tumor characteristics before surgery and could more accurately locate renal tumors during surgery. Therefore, there were significantly more patients who underwent LPN in the MRALN group than in the non‐MRALN group (82% vs 46%, *P* < .001). In addition, we demonstrated that MRASN can effectively improve the efficiency of LN. MRALN significantly shortened OT by approximately 38.3%, decreased WIT by approximately 38.4% and reduced EBL by approximately 66.2% compared with the corresponding outcomes in the control group. Although there were no statistical differences in preoperative or postoperative creatinine between the MRALN and non‐MRALN groups, the postoperative creatinine value of the MRALN group was lower than that of the non‐MRALN group. The main reason is that there is no early method to monitor split renal function, and the compensatory effect of residual nephron and healthy kidney offset the difference in creatinine values between the two groups.

At present, the mechanism of APF is not clear. It has been reported that it may be related to the inflammatory microenvironment, metabolic syndrome (MetS), autoimmune response, idiopathic fibrosis, and so on.^19^, ^20^ Naoko Kawamura et al^21^ proposed that the occurrence of APF may lead to difficulty of renal decapsulation during dissection and then increase EBL (compared with non‐APF EBL, 861 vs 528 mL, *P* < .01). Neil J. Kocher et al^22^ conducted research on the effect of APF occurrence on perioperative outcomes and found that APF was associated with increased OT duration (*P* = .005). Zine‐Eddine Khene et al^23^ reported that the occurrence of APF can lead to a higher risk of conversion to radical nephrectomy (RN) or to open partial nephrectomy (OPN). In our study, MR technology was used for the first time to display the APF area so that the surgeons were able to identify the surrounding conditions of renal tumors before the operation, which was conducive to the correct selection of operation methods, surgical risk assessment, and operation plan formulation.

In recent years, a large number of advanced technologies, represented by 3D modeling, 3D printing, VR, AR, and surgical robots, have been developing rapidly in the field of surgery, influencing surgeons’ thinking, operation, and habits.^24^, ^25^, ^26^, ^27^, ^28^ Before surgery, such technology can enable surgeons to evaluate patients’ imaging information in the most natural way—that is, intuitively, stereoscopically and comprehensively—greatly reducing the difficulty of identifying the complex spatial structure relationship of tumors and significantly shortening the preoperative learning cycle of surgeons. During the operation, it is helpful for the accurate localization and resection of the tumor and matching the 5G network with MRASN to achieve high‐throughput computing analysis of holographic MR stereo images and 4K‐resolution cloud video live broadcast so that experts in different places can share and interact with the surgeon in real time, thereby realizing digital surgery and telemedicine platforms. Venkata et al^29^ proved the application value and accuracy of MR in remote consultation, allowing the surgeon to video‐conference with experts in remote areas.

There were a few limitations to current MRASN. First, the sample size of this study was relatively small. The conclusions of this research need to be further studied in a larger data set. Furthermore, more surgeons should participate in the subjective feedback of MRASN application value. Second, we have not quantified the accuracy of MRASN. Third, regarding MRASN registration, when the renal artery is blocked, the kidney will undergo some soft tissue deformation, which will lead to some errors between the MR image and kidney registration. Fourth, MR real‐time and dynamic registration cannot be realized at present. Only when the surgeons need accurate judgment can they adjust the MR holographic field of vision at any time and undergo MR register judgment after the laparoscopy video stream is stopped. Finally, most laparoscopic windows do not have TiePro display function, which is similar to the robotic surgery window, so we need to fuse in the new monitor (with video capture card). However, HoloLens MR‐HMD itself has this function. We believe that with the development of surgical navigation, these problems will be solved gradually.

## 5 CONCLUSIONS

Mixed‐reality‐assisted laparoscopic nephrectomy can effectively improve the number of successful implementations of LPN, greatly shorten OT and WIT, and reduce EBL. MRASN has a certain clinical application value in operative plan formulation, intraoperative navigation, remote consultation, teaching guidance, and doctor‐patient communication.

## CONFLICT OF INTEREST

The authors declare that they have no conflict of interest.

## AUTHOR CONTRIBUTIONS

Guan Li: project development, data collection and management, data analysis, and manuscript writing. Zhiqiang Cao and Jie Dong: project development and data collection. Guangming Lu: project development and manuscript editing. Jinbao Wang: data analysis and manuscript editing. Xin Zhang: supervision and manuscript editing. Dongbing Cao: project development, supervision, and manuscript editing.

## Data Availability

The data used to support the findings of this study are available from the corresponding author upon request.
